# Implications of HIV persistence and pathogenesis in microglia

**DOI:** 10.1097/COH.0000000000001044

**Published:** 2026-06-24

**Authors:** Nanouk Zuidmeer, Jori Symons, Monique Nijhuis

**Affiliations:** a Translational Virology, Department of Medical Microbiology, University Medical Center Utrecht, Utrecht, The Netherlands; b HIV Pathogenesis Research Unit, Department of Molecular Medicine and Haematology, School of Pathology, Faculty of Health Sciences, University of the Witwatersrand, Johannesburg, South Africa

**Keywords:** brain, HIV pathogenesis, HIV persistence, microglia, neuroinflammation

## Abstract

**Purpose of the review:**

This review summarizes recent advances in understanding HIV infection of the brain, focusing on viral persistence in microglia and associated pathogenesis during viremia and viral suppression. We highlight key findings in microglial research that inform the next steps toward therapeutic strategies and cure approaches addressing the brain as a viral reservoir.

**Recent findings:**

Microglia can contain intact, replication-competent inducible proviruses, even during antiretroviral therapy (ART). The microglial chromatin context and HIV integration site landscape during HIV infection are reportedly distinct from peripheral lymphocytes. Upon infection, both infected and bystander microglia exhibit pro-inflammatory and senescent phenotypes that closely resemble those observed in aging microglia and play a central role in neuropathogenesis. ART mitigates these effects but does not fully restore to levels observed in HIV-uninfected conditions.

**Summary:**

Microglial HIV infection and persistence of this reservoir play a central and detrimental role in HIV pathogenesis in the brain. Given their role in neurodegeneration during HIV infection, closer characterization of the microglial (latent) reservoir and careful monitoring of neurodegeneration during treatment and cure interventions are essential. Treatment and cure strategies should prioritize minimizing viral transcription and protein expression within the brain reservoir to mitigate neuroinflammation and neuropathogenesis.

## INTRODUCTION

CD4^+^ T cells are the main target for HIV in the periphery, but myeloid cells are also indicated to play a significant role in HIV persistence and pathogenesis in complex tissues, like the brain. Despite antiretroviral therapy (ART), HIV can persist in these tissue reservoirs, presenting a major barrier to HIV cure.

The brain constitutes a unique and immune-privileged compartment that is shielded from the periphery by the blood–brain barrier. Within this unique microenvironment with limited lymphocyte infiltration, HIV can establish a reservoir in microglia, the brain resident immune cells [1]. While ART effectively blocks viral replication, HIV can be persistently transcriptionally active, producing viral particles and (neurotoxic) viral proteins even during viral suppression [2]. Notably, persistent infection of the central nervous system (CNS) has significant clinical consequences ranging from HIV-associated dementia (HAD) in the absence of ART to HIV-associated neurological impairment (HAND) [3,4].

The unique and vulnerable microenvironment in the brain makes for a complex and enigmatic tissue reservoir. This review highlights recent studies that give insight into HIV persistence and pathogenesis in microglia. We summarize key findings, methodologies, and consensuses to enlighten the next steps toward treatment and cure that comprehensively includes the brain's viral reservoir. 

**Box 1:**
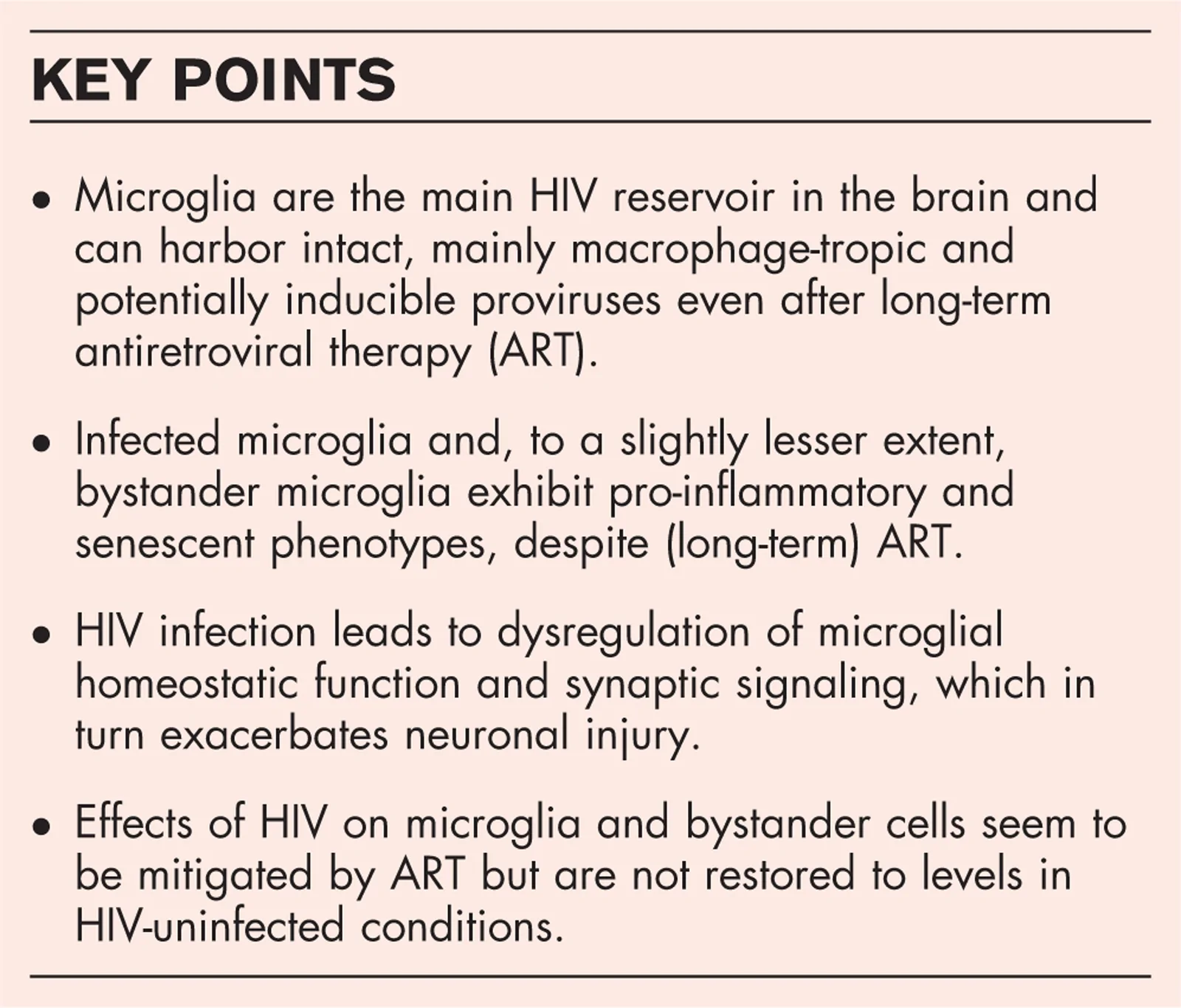
no caption available

## MICROGLIA ARE LONG-LIVED AND CRITICAL FOR MAINTAINING BRAIN HEALTH

Microglia are the most abundant myeloid cells in the brain and represent 5–20% of the adult brain cells. They originate from hematopoietic progenitors in the yolk sac and migrate into the CNS during early embryonic development [5,6]. Microglia are highly heterogenic and long-lived, with a calculated median renewal rate of ~30% per year and an estimated average lifespan of 4.2 years, though some microglia can last more than two decades [7]. Microglia are critical in maintaining brain homeostasis, protecting neuronal health and responding to pathological damage in the brain [8]. As the brain's key immune effector cells, microglia express many immune receptors and pattern-recognition receptors as well as many pro- and anti-inflammatory factors [8,9]. Notably, microglia do not only surveil to eliminate pathogens, but also eliminate waste such as dead cells, redundant or damaged synapses, and protein aggregates, therefore playing a vital role in neurogenesis and regulation of synaptic density and plasticity [8]. Microglial function is closely intertwined with that of other glial cells (e.g. astrocytes and oligodendrocytes) and neurons, through intensive cell-cell crosstalk and their involvement in myelination [8]. Due to their extensive functions, microglia are vital players in maintaining brain health, and their dysregulation is associated with and functionally contributes to the pathogenesis of diverse neurological disorders (e.g. Alzheimer's disease) and brain aging [10,11].

## MICROGLIA AS MAIN HIV RESERVOIR IN THE BRAIN

While the brain is a highly sequestered microenvironment, viral spread into the CNS happens within weeks after HIV infection [12]. CNS invasion likely occurs through transmigration of infected cells or invasion of cell-free virus past the blood brain barrier [13,14]. Microglia, like other myeloid cells, express the HIV binding receptor CD4 at low levels and co-receptors, CCR5 and CXCR4. While T cell (T)-tropic viral strains require high CD4 expression for viral entry, the so-called macrophage(M)/T-tropic strains can also infect cells with low CD4, including microglia [15].

Within the brain, microglia are well reported to constitute the main cellular reservoir for HIV [1], though other cell types have been implicated in the CNS HIV reservoir [16]. They were identified as dominant HIV reservoir already in the 1990s, through the detection of multinucleated giant cells and viral gp41+ microglia in brain slices of people with HIV (PWH) with acquired immunodeficiency syndrome (AIDS) encephalitis (HIVE) [17–20]. The distribution of HIV-infected cells throughout encephalitic tissue was heterogenic and focalized [21]. Later, detection of HIV DNA [22] and RNA [18] in microglia in HIV encephalitic tissue robustly confirmed this cellular compartment to be the main target of HIV in the brain.

## PERSISTENCE OF HIV IN MICROGLIA

More recent detection of viral DNA and RNA in microglia from *postmortem* brain tissues has further highlighted the role of microglia as viral reservoir during viral suppression by ART [23^▪▪^,^24^–^26^,^27^,28^▪^]. Notably, total HIV DNA was found to be present at comparable levels in brain tissue of untreated and virally suppressed (VS) PWH [26,29], reflecting a stable viral reservoir that persists despite therapy. In VS PWH viral RNA was, when detected, localized in infrequent foci [24]. Here, we discuss in more detail the microglial reservoir and its characteristics during ART.

### INTACT HIV proviral DNA in microglia

In peripheral T cells, the vast majority of integrated HIV DNA is defective, with only ~3% of infected cells carrying intact proviruses [30]. In monocytes, up to 40% of proviral DNA is intact in VS PWH [31,32]. By extension, solely the detection of total HIV DNA in microglia does not resolve whether these cells constitute an intact viral reservoir. Addressing this distinction requires assays capable of discriminating and quantifying defective and intact proviral DNA, such as the intact proviral DNA assay (IPDA) [33,34]. Using this assay on *postmortem* brain tissue of PWH, several recent studies revealed that total and intact proviral DNA can be detected across brain tissue of both viremic and VS PWH [25,26,27^▪^]. Recently, it was shown that intact and defective proviruses persist across multiple brain regions (frontal white matter [FWM], basal ganglia, and cerebellum) even after long-term ART [25]. Viral suppression does not seem to alter the distribution of total HIV DNA throughout the tissue. However, among the three regions studied by Angelovich *et al.,* FWM harbored the most intact proviral DNA and FWM showed to be the largest regional reservoir of intact proviral DNA during long-term ART [25]. While these bulk tissue analyses implied a myeloid origin of these intact proviruses, more direct evidence of intact microglial reservoirs was recently provided through IPDA analysis of CD11b+ microglia cells from fresh *postmortem* brain tissue [27^▪^]. The study demonstrated 13–15% intact proviral DNA across three separate brain regions (frontal lobe, subventricular zone and occipital lobe) of one VS individual, similar to findings in whole brain of VS PWH by Cochrane *et al.* (15.5% intact) [26]. Moreover, phenotypic characterization of full-length *env* genes amplified from this individual showed that ~80% encoded for M/T-tropic viruses capable of infecting monocyte derived microglia-like cells (MDMi) [27^▪^]. These three studies collectively show the presence of intact and potentially replication-competent proviral DNA in the brain, despite (long-term) viral suppression.

### Microglia as replication-competent and inducible reservoir

Until recently, it was not clearly demonstrated whether the intact proviruses harbored by microglia are replication-competent and inducible or in a deep latent state. In 2023, one unique study showed evidence of viral outgrowth from primary microglia of *postmortem* brain tissue of VS PWH [35]. Notably, replication-competent virus could be recovered following *ex vivo* stimulation of isolated primary microglia, capable of infecting both microglia and peripheral CD4^+^ T cells, and phylogenetically distinct from those from peripheral sources [35]. Uniquely, the data support the presence of replication-competent and inducible HIV in microglia, but do not define the size or relative contribution of the microglial reservoir compared with other compartments.

Inducibility and replication-competence are not only dependent on proviral intactness, but also on integration sites (IS) [36,37]. Although HIV preferentially integrates in open chromatin structures in both CD4+ T cells and microglia [38], the (epi)genomic landscape differs between these cellular reservoirs [39]. In microglia of PWH with HIVE, a slight but significant shift was found in the proportion of the host genome found in euchromatin compared to non-HIVE HIV+ and HIV− microglia. Notably, immune-related loci were generally de-repressed while homeostatic and neuronal support-associated loci were generally repressed in HIVE and to a lesser extent in non-HIVE HIV+ microglia [39]. In nonneuronal nuclei, IS were more often localized in intergenic regions, and accordingly, had higher rates of IS in heterochromatin, while still overall favoring integration in euchromatin compared to peripheral lymphocytes [39]. This may be linked to decreased expression of *LEDGF* and *CPSF6*, cellular factors which in T cells mediate integration site selection in euchromatin. In *postmortem* brain tissue of VS people with HIV (PWH) ~0.5% of microglia were detected containing cell-associated HIV RNA and DNA in euchromatin regions [40^▪^]. Collectively, these observations indicate that chromatin structures and IS landscapes in microglia differ slightly from peripheral lymphocytes, with similar rates of integration in euchromatin but more integration in intergenic regions in microglia.

Summarizing, microglia are long-lived cells, resistant to cell death, and can harbor intact [25,26,27^▪^], mostly M/T-tropic [27^▪^], and possibly inducible [35] proviral DNA even after long-term ART. While this makes them potentially a very persistent reservoir, longitudinal brain data is unavailable, thus proviral decay over long-term ART is hard to study. However, similar patterns and consistent reports of HIV DNA detection in viremic PWH and PWH on long-term ART might suggest slow decay. More research is desperately needed to examine the viral dynamics of the microglial reservoir, and how they compare to other (peripheral) cellular reservoirs. Exploring viral compartmentalization, decay and productivity in the brain both without and during viral suppression would give better insight in the possible interventions needed to silence and eliminate the microglial reservoir.

## HIV PATHOGENESIS IN MICROGLIA

Microglial activation is highly heterogenic and dynamic, with different activation signatures between injuries and diseases. Accordingly, their functional and -omics features differ significantly depending on the pathological context. Here, we review the current paradigms of microglial pathogenic signatures during HIV infection.

### HIV infection induces a pro-inflammatory state in microglia

Generally, microglia display a pro-inflammatory phenotype upon HIV infection *in vivo* and *in vitro*[23^▪▪^,28^▪^,^39^,40^▪^,^41^^▪▪^,^42^^▪▪^,43^▪^]. Single nuclei transcriptome analysis of *postmortem* brain tissue of PWH with a history of treatment with ART (one third of participants were on recent suppressive ART) revealed an upregulation of type II interferon (IFN)-response and -signaling pathways, as well as responses and defense against virus in microglia [23^▪▪^]. Moreover, Yang *et al.* were able to identify three gene modules corresponding to 3 groups of microglia, which encapsuled downregulation of homeostatic genes, upregulation of pro-inflammatory genes and upregulation of hypoxia-related pathways in combination with lipid accumulation. Interestingly, lipid droplet accumulation in microglia have been characterized in a neuro-HIV context and only appeared in PWH in this study [23^▪▪^]. Additionally, *in vitro* infected microglia exhibited a pro-inflammatory phenotype as well, characterized by robust increase in NF-κB expression upon infection [41^▪▪^,43^▪^] and exposure to viral proteins [44–46]. IFN-mediated, tumor necrosis factor (TNF)/NF-κB, and interleukin (IL)-6/JAK/STAT pathways were identified as key drivers of inflammation in microglia during HIV infection [42^▪▪^,43^▪^]. Likewise, IL-1β, TNF-α, CCL2, and type I IFN-responses were increased in induced pluripotent stem cell-derived microglia (iPSC-M) after HIV infection [47^▪^]. Inflammatory pathways were upregulated within hours after infection in iPSC-M, a pattern distinct from infected monocyte derived macrophages (MDMs) [43^▪^]. Notably, inflammatory responses *in vitro* were similar compared to those previously identified in brain tissue of people with HAND [43^▪^].

Recently, Mason *et al.* reported in a preprint that microglia do not only exhibit activation upon HIV infection, but HIV infection also drives cellular senescence [42^▪▪^]. Analysis of single-cell and bulk transcriptomic patterns in *ex vivo* iPSC-M HIV infection and *in vivo* simian immunodeficiency virus (SIV) infected rhesus macaque models revealed cellular senescence with strong parallels to transcriptomic patterns of aging, characterized by p53 pathway activation, downregulation of proliferative genes and upregulation of inflammatory genes. Critically, these senescent signatures were observed in not only (productively) HIV/SIV-infected, but also uninfected bystander microglia [42^▪▪^]. Likewise, in *postmortem* brain tissue of VS PWH, several subclusters of microglia revealed heterogenic transcriptomes related to mainly microglial activation (e.g. mitochondrial, proliferation and ribosomal genes) and inflammation (e.g. IL1B, IFN-signaling and cyto- and chemokine genes), but also senescence [40^▪^]. While perturbations of microglial homeostasis and inflammation across studies supports the paradigm that bystander microglia are severely impacted by HIV infection, HIV infected microglia have distinguishable patterns from uninfected bystanders. HIV RNA+ microglia from brain tissue (only ~0.5% of microglia) displayed a more inflammatory phenotype than bystanders [40^▪^]. Similarly, in organoid-derived iPSC-M upregulation of cytokine and IFN-γ signaling and downregulation of neurosupportive pathways were detected in HIV RNA+ microglia compared to bystanders [48^▪▪^].

While ART diminished these cellular senescence and pro-inflammation patterns in SIV-infected animals, some transcriptional patterns in microglia persisted despite viral suppression and do not fully restore to homeostasis [42^▪▪^]. This residual inflammation upon viral suppression was also reported in iPSC-M containing brain organoids [41^▪▪^,^48^^▪▪^]. In addition, deregulation of immune and inflammatory pathways in microglia from *postmortem* tissue of VS PWH confirm the persistent disturbance of microglia homeostasis despite viral suppression [23^▪▪^,40^▪^]. In a recent preprint, we uniquely report through RNA-seq data from *post mortem* brain tissue, that viral suppression mitigates microglial dysregulation in VS PWH as compared to viremic PWH, but residual homeostatic, synaptic and metabolic disruption and neuroinflammation persist in VS PWH compared to HIV-uninfected individuals [28^▪^,^49^].

Evidently, microglial action is impaired upon infection with HIV, which can contribute to neurodegeneration through release of neurotoxic viral proteins (e.g. Tat [50]), neurotoxins and pro-inflammatory cytokines (e.g. IL-8, IL-6, IL-1β, and TNF-α [51]). These factors can in turn induce astrocyte differentiation and apoptosis, as well as alter homeostatic neurogenesis [52]. If left untreated, this excessive neuroinflammation and impaired microglial functioning can result in brain structure and function loss [53,54]. Altogether, these data highlight the critical contribution of microglia to HIV-associated neuropathogenesis. The pro-inflammatory and senescent phenotype induced by HIV infection in both infected and bystander microglia severely impact neuronal health, neurodegeneration and general brain health, which does not seem to be fully restored by ART. For this reason, it is critical that further research focusses on fully exploring the effect of ART and cure strategies on microglial health and the subsequent impact on brain health and neurocognitive decline.

### Future directions

Much remains unknown about the specific effects and impact of microglial infection and pathogenesis. Therefore, this review includes several studies that are currently available as preprints and have not yet undergone peer review. While these findings remain provisional, they nevertheless provide important insights in our understanding of the microglial HIV reservoir and steer approaches for CNS-specific cure strategies. These approaches are called for, as the microglial reservoir dynamics might differ significantly from peripheral reservoirs, given their intrinsic resistance to HIV-induced cell death [55] and the brain's immune-seclusion with limited lymphocyte infiltration.

Additionally, it is vital that the detrimental impact of HIV on the brain is diminished as much as possible. The mitigation of neuroinflammation by ART suggests a substantial role of viral suppression in protection against worsened impairments. Therefore, it is of critical importance that neural and neurological damage is closely monitored during ART interruption and latency reversal interventions in clinical trials. Moreover, viral transcription and (neurotoxic) protein production should be minimized as much as possible, as to not induce (or worsen) neuroinflammation. To achieve this, latency promoting agents (LPAs) could potentially play a significant role by inducing the reservoir into a (deeper) latent state and reducing transcription. On the other hand, latency reversal agents (LRAs), by inducing viral transcription, may exacerbate neuroinflammation, as reviewed by Nühn *et al.* [55].

We recommend pursuing efforts to a biomarker that allows for monitoring of brain health during treatment and cure trails (see Box 1). In addition, we emphasize the necessity of taking the brain HIV viral reservoir (dynamics) into account and urge close monitoring of neurodegeneration in any LRA, ART interruption or cure study. Critically, we highlight the importance of both *in vitro* and *in vivo* examinations of microglial (latent) reservoirs and their response upon treatment and cure strategies [56].

Box 1 **Recommendations**

**Table:** 

- Assess the role of viral replication and production in neurodegradation and the possible role for LPAs - Monitor brain health and neuroinflammation during cure trails, ART interruptions and treatment, specifically when using LRAs ○ Pursue efforts to find biomarkers for neurodegradation and -inflammation in accessible samples ○ Utilize -omics platforms and phenotypical assessments to find HIV-specific perturbation patterns - Consider and explore HIV subtype-dependent differences - Utilize *in vitro* models to characterize latency in microglia and inducibility of these latent proviruses - Characterize replication competence of microglial reservoir in larger cohorts
For a comprehensive understanding of HIV infection in microglia and its consequences on neurocognitive and neuronal impairment *in vivo, in situ* and *in vitro* models will all play a vital role.

### Concluding remarks

**FIGURE 1:**
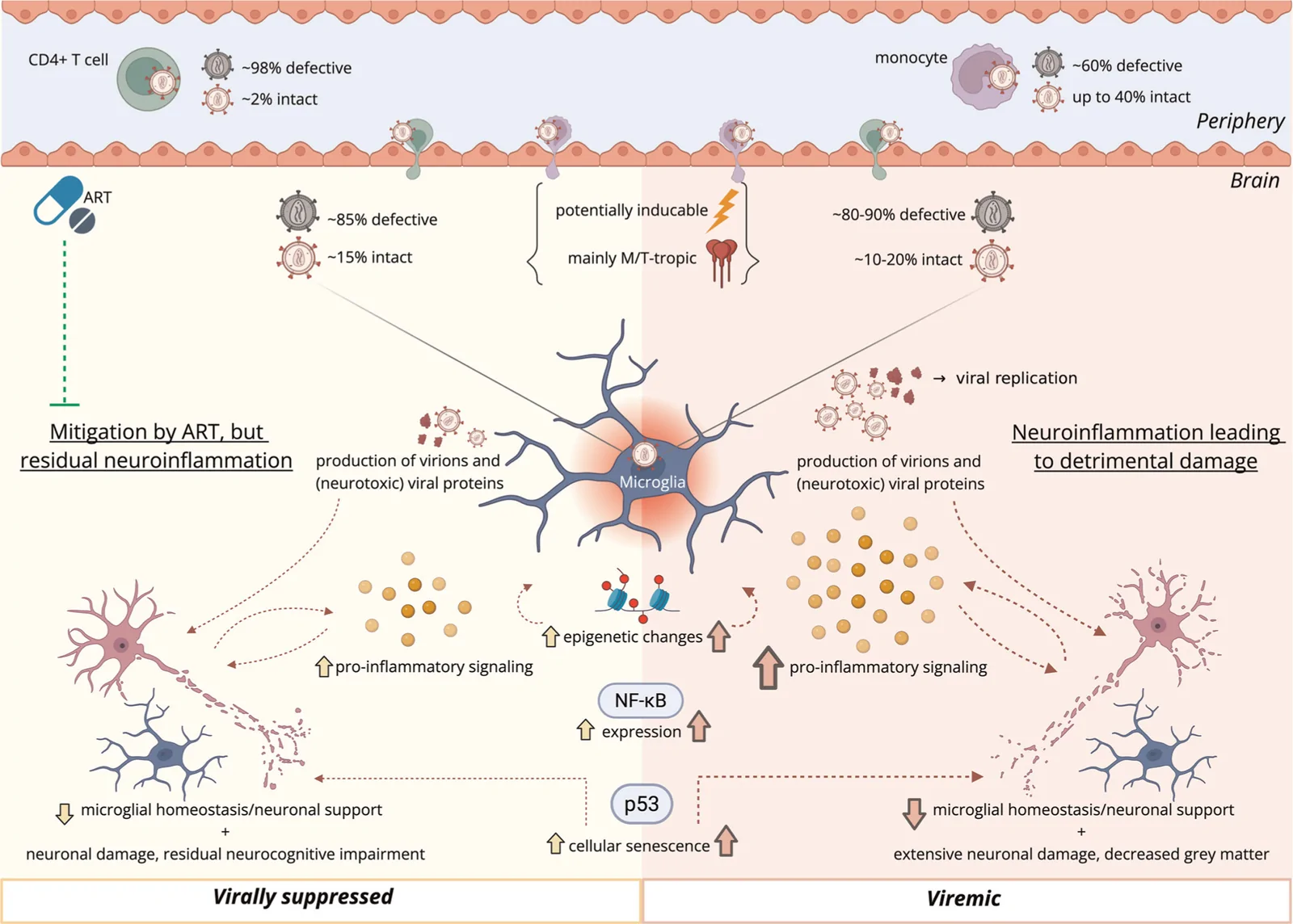
Overview of the current paradigms of microglial HIV infection, persistence and pathogenesis, during viremia and viral suppression. Created in BioRender. Zuidmeer N. (2026). https://BioRender.com/72gm84y.

Microglia are the main HIV reservoir in the brain and pose a great challenge to HIV treatment and cure. Considering the most impactful recent studies, it is evident that microglia can harbor intact and replication competent provirus, regardless of viral suppression. HIV infection of microglia induces pro-inflammatory and senescent phenotypes in microglia and uninfected bystander cells, perturbations which seem to be mitigated but not restored upon viral suppression. Impaired microglial homeostasis and neuroinflammation will lead to detrimental harm to neurons, manifesting as mild to severe neurocognitive impairments in (VS) PWH (Fig. 1).

## ACKNOWLEDGEMENTS

*None.*

### Financial support and sponsorship

*Part of the data presented in this review generated with support of grants received from Health-Holland (LSHM20012-SGF, LSHM19100-SGF and LSHM19101-SGF), Spiral Integrated intervention to achieve durable HIV control in diverse populations; (KICH2.V4P.AF23.001) and the Aidsfonds (P-66401 and P-22604).*

### Conflicts of interest

*There are no conflicts of interest.*

## REFERENCES AND RECOMMENDED READING

Papers of particular interest, published within the annual period of review, have been highlighted as:

▪ of special interest
▪▪ of outstanding interest
